# Effect of anacardic acid against echinococcosis through inhibition of VEGF-induced angiogenesis

**DOI:** 10.1186/s13567-019-0621-7

**Published:** 2019-01-14

**Authors:** Miaomiao Yuan, Xiaoxia Song, Wei Lv, Qi Xin, Li Wang, Qi Gao, Guochao Zhang, Wenzhen Liao, Sen Lian, Tao Jing

**Affiliations:** 1 School of Basic Medical Sciences, Lanzhou University, Lanzhou, 730000 China; 2 Cancer Research Institute, Guangdong Provincial Key Laboratory of Cancer Immunotherapy, Guangzhou Key Laboratory of Tumor Immunology Research, School of Basic Medical Sciences, Southern Medical University, Guangzhou, 510515 China; 3 Department of Traditional Chinese Medicine, People’s Hospital of Qinghai Province, Xining, 810007 China; 4 Department of Nutrition and Food Hygiene, Guangdong Provincial Key Laboratory of Tropical Disease Research, School of Public Health, Southern Medical University, Guangzhou, 510515 Guangdong China; 5 Department of Biochemistry and Molecular Biology, Guangdong Provincial Key Laboratory of Biochip, School of Basic Medical Sciences, Southern Medical University, Guangzhou, 510515 Guangdong China

## Abstract

Echinococcosis is a zoonotic infection caused by cestode species of the genus *Echinococcus,* with limited treatment options. It is urgent to develop new anti-hydatid agent. In this paper, we reported anacardic acid (AA), a natural product isolated from the Brazilian cashew-nut shell liquid, which presented a high activity against metacestodes of *Echinococcus multilocularis* (*E. multilocularis*) and *Echinococcus granulosus* sensu stricto (*E. granulosus* s.s.) in vitro and in vivo. AA exerted a better efficacy on *E. granulosus* s.s. protoscoleces and *E. multilocularis* metacestodes than that of albendazole (ABZ) and dihydroartemisinin (DHA) in vitro, and an inhibition on the growth of *Echinococcus* metacestode as effective as ABZ in vivo. Moreover, we also found that one of the mechanisms of AA against *Echinococcus* could be the suppression of angiogenesis on/in the metacestode mass through inhibiting vascular endothelial growth factor (VEGF)—induced signalling pathways. This work finds that AA is a new promising potential candidate drug for echinococcosis treatment.

## Introduction

Echinococcosis is a cosmopolitan zoonotic disease which is acquired via infection with members of the genus *Echinococcus* at the larval stage. Human is mainly infested by two types of echinococcosis: cystic echinococcosis (CE) caused by *E. granulosus* sensu lato (s.l.) which is distributed around the world, whose primary definitive host is dogs, while alveolar echinococcosis (AE), caused by *E. multilocularis*, is generally confined to the northern hemisphere with foxes acting as the main hosts. Severe and even fatal consequences occur if eggs are ingested by human and other hosts [1, 2]. Currently, surgery is the basis of treatment for early AE, but patients not suitable for surgery and those who have had surgical resection of parasite lesions must be treated with chemotherapy. The treatment options for CE are: drugs, surgery and percutaneous sterilization [2, 3]. The primary chemotherapeutic drugs used for echinococcosis are benzimidazole carbamate derivatives, such as ABZ and mebendazole (MBZ), which inhibit microtubule polymerization by selectively binding to parasite tubulin [4], but these drugs are parasitostatic rather than parasitocidal [4]. Besides, there are several other significant limitations with this current treatment, including: (i) cases where these drugs are ineffective, and (ii) reported toxicity [5]. Therefore, it is critically urgent to identify new drugs for the more effective treatment of echinococcosis.

As is well-known, in alveolar echinococcosis, the metacestodes are continuous and asexual proliferation by exogenous budding, resulting in the tumor-like, infiltrative growth of the parasite lesion [6]. Nowadays, it is reported that many antitumor drugs, including cyclosporine [7], osthole [8], doxorubicin [9], imatinib [10], 2-methoxyestradiol [11], bortezomib [12], and mefloquine [13] present a quite high anti-echinococcosis activity. AA, a natural product isolated from the Brazilian cashew-nut shell liquid, features a convenient salicylic acid system and a long side chain at the 6-position, in which a double bond is found at C-8 in the monoene, diene and triene components [14]. This compound has been attracting considerable attention due to its diverse biological effects such as: (1) anti-microbial activity [15], (2) anti-fungal activity [16], (3) anti-tumoral activity [17], (4) anti-parasitic activity [18], (5) anti-insectival activity [19], (6) gastroprotection [20], and (7) inhibition of enzymes such as lipoxygenase [21], tyrosinase [22], and histone acetyltransferases [23]. However, the effect of AA on echinococcosis still remains unknown. Thus, the present experiment was design to demonstrate the effect and explore the possible mechanisms of AA against echinococcosis.

## Materials and methods

### Biochemicals and drugs preparation

AA was purchased from Selleckchem. All culture media were purchased from Gibco-BRL and the other reagents were purchased from Sigma. AA, ABZ and DHA were dissolved into DMSO at a final concentration of 40 mM and sterilized with 0.22 μm filter membrane.

### Preparation of *E. granulosus* s.s. protoscoleces for in vitro experiment

Protoscoleces of hydatid cysts were removed aseptically from a naturally infected sheep from a slaughterhouse located in Xining, Qinghai Province, China. The genotype of protoscoleces from sheep and germinal cells from secondary infected mice was identified as *E. granulosus* G1 strain [24]. An in vitro culture of *E. granulosus* s.s. protoscoleces were maintained as previously described [25]. Briefly, the harvested protoscoleces were washed five times with saline, transferred to T25 culture flasks containing culture medium (Dulbecco’s minimal essential medium (DMEM), 2 mM glutamine, 12 mM HEPES, 100 U/mL of penicillin, and 100 U/mL of streptomycin) supplemented with 10% fetal bovine serum (FBS), and incubated in an upright position in an incubator at 37 °C and 5% CO_2_. Finally, the protoscoleces were cultured under this condition 3 days for further use.

### Efficacy of AA against *E. granulosus* s.s. protoscoleces in vitro

Treatments were performed using 24-well tissue culture plates containing 100 protoscoleces/well and 1 mL of culture medium without FBS and phenol red. AA at a serial concentrations of 0.5, 1, 2, 4, 10, and 20 μM was used for the experiment, and ABZ (40 μM) and DHA (40 μM) served as a positive controls. 0.1% DMSO was used as a negative control and its viability was defined as 100%. The mortality of the protoscoleces was assessed using the trypan blue exclusion test and visualized on an inverted microscope at 100× magnification [25]. The effect of different drug treatments on the morphology and structural integrity of protoscoleces was visualized at 100× magnification on day two before trypan blue staining. Each experiment was repeated twice. EC_50_ values were calculated in OriginPro 8.

### Preparation of *E. multilocularis* metacestodes for in vitro experiment

In vitro cultivation of *E. multilocularis* metacestodes was carried out as previously described [26]. Briefly, *E. multilocularis* metacestodes were obtained from the infected BALB/c mice via intraperitoneal injection of minced metacestode tissue. After three months, the infected BALB/c mice were euthanized, and metacestodes were removed from the peritoneal cavity and cut into tissue blocks of about 0.5 cm^3^ under a fully sterile condition. After washing twice with DMEM, three or four tissue blocks were placed in cell culture flasks which were pre-cultured with HepG-2 cells containing 40 mL of DMEM, 10% FBS, 100 U/mL of penicillin, 100 U/mL of streptomycin, 12 mM HEPES, and 2 mM glutamine. The tissue blocks were kept in tightly closed culture flasks and incubated at 37 °C in 5% CO_2_, with the medium being changed twice a week. Finally, these metacestodes were used for in vitro drug assays as described below.

### Efficacy of AA against *E. multilocularis* metacestodes in vitro

Following 8-weeks of culture, the vesicles with a diameter between 1 and 5 mm were harvested from the *E. multilocularis* co-cultures and washed three times in serum-free medium. Then, approximately 20 vesicles in 1 mL of DMEM culture medium without FBS and phenol red were added into 24-well plates. AA with serial concentrations of 0.5, 1, 2, 4, 10, 20 and 40 μM was respectively added to the cultures. An equal amount of 40 μM ABZ and 0.1% DMSO were added as controls. All the cultures were incubated at 37 °C with 5% CO_2_. Finally, supernatants of the culture media were collected on day 7 following drug treatments and centrifuged at 10 000* g* for 10 min at 4 °C, then stored at −20 °C for *E. multilocularis* alkaline phosphatase (EmAP) activity assays [27]. All experiments were performed in duplicates.

Quantitative assessment of EmAP activity in the culture supernatant was performed as described by Stettler et al. [28]. EmAP was tested via an AP activity assay kit purchased from Beyotime, China. Briefly, 50 μL culture supernatant, mixed with 50 μL of alkaline phosphatase chromogenic substrate, then it was incubated for 30 min at 37 °C, and finally 100 μL stopping buffer was added to each well of the 96-well plates. The values of OD at 405 nm were read on an enzyme-linked immunosorbent assay reader.

At 7 days of post-treatment with 0.5 μM AA, metacestodes were processed for scanning electron microscopy (SEM) as described previously [29]. Briefly, metacestodes were washed twice with PBS, placed into 2.5% glutaraldehyde at 4 °C over night for pre-fixation followed by post-fixation in 2% OsO_4_ for 2 h at room temperature. Then they were washed twice with PBS, dehydrated in serially increasing concentrations of ethanol for 10 min and immersed into isoamyl acetate for 1 h at room temperature. The specimens were then sputter coated with gold and observed under SEM after dehydration via a critical point drying technique.

### Efficacy of AA against *E. granulosus* s.s. metacestodes in vivo

Protoscoleces of hydatid cysts were removed aseptically from a naturally infected sheep from a slaughterhouse located in Xining, Qinghai Province, China. The female C57BL/6 mice (*n* = 20) infected with *E. granulosus* s.s. protoscoleces for 18 weeks were randomly divided into four groups of 5 animals each. These animals orally received the following treatments respectively every day for 6 weeks: group 1 (non-infected) received 0.4 mL honey/PBS (1:1 v/v); group 2 (infected-no treatment, control) received 0.4 mL honey/PBS (1:1 v/v); group 3 (infected + ABZ treatment, positive control) received 100 mg/kg ABZ in 0.4 mL honey/PBS (1:1 v/v); and group 4 (infected + AA, experimental group) received 100 mg/kg AA in 0.4 mL honey/PBS (1:1 v/v). After the treatments, mice were anesthetized with an intraperitoneal injection of 10% chloral hydrate (3.5 mL/kg), and blood samples via ocular sinus were collected before euthanasia. Subsequently, the blood samples were centrifuged at 3000 *g* for 15 min at 4 °C, incubated at 37 °C for 1 h, and finally the sera were harvested and stored at −20 °C for IL-4 detection. The level of IL-4 was determined with Enzyme-linked immunosorbent assay (ELISA) following the manufacturer’s instructions. In addition, all mice were sacrificed by cervical dislocation, and the cysts in the peritoneal cavity were isolated and weighed. The efficacy of the treatments was assessed based on mean cyst weight [24]. The ultrastructural changes of the germinal cells were observed under SEM. All data were analyzed by one-way ANOVA.

### Efficacy of AA against *E. multilocularis* metacestodes in vivo

Female BALB/c mice were purchased from the Laboratory Animal Center of Lanzhou University, and housed in a temperature-controlled daylight/night cycle room with food and water ad libitum. For animal infection, *E. multilocularis* metacestodes were initially isolated from euthanized BALB/c mice which had been intraperitoneally infected with *E. multilocularis* metacestodes. At 12 weeks post-inoculation, mice were euthanized and metacestode tissues were removed from the peritoneal cavity, minced, and suspended in DMEM under sterile conditions [30]. All female BALB/c mice were infected intraperitoneally with 300 μL of the metacestode suspension and randomly separated into three treatment groups of 5 mice each. After 18 weeks post-infection, the mice received the same treatment with *E. granulosus*-infected mice. Treatments were repeated daily for 6 weeks, and then blood samples were processed, tested in the same way as *E. granulosus*-infected mice mentioned above, and finally used for IL-4 and VEGF detection. The metacestode tissues from all mouse models were collected to measure the total wet weights of the metacestodes from each group. Meanwhile, the metacestode tissues were fixed, sectioned, and stained with hematoxylin and eosin (H&E) for microscopic examination. In addition, *E. multilocularis* metacestodes from the controls and the experiments were isolated, fixed with 10% formaldehyde, and embedded in paraffin for immunohistochemical analysis, in which anti-CD34 antibodies were used to stain blood vessels. The images were quantified and analyzed by Image J software. All data were analyzed by one-way ANOVA.

### Cytotoxicity of AA on Chang liver cells and HepG2 cells

Chang liver cells or HepG2 cells were seeded in 96-well plates with a density of 1 × 10^4^ cells/well, and cultured for 24 h. The cells were treated with serial concentrations (0.5, 1, 2, 4, 10, 20, 40, 80, 120, 160 μM) of AA for 48 h before being assayed for cell viability by MTT assay. The absorbance values of OD at 490 nm were read using an ELx800 absorbance microplate reader [31]. The IC_50_ values were calculated in OriginPro 8.

### Ethics statement

BALB/c mice and C57BL/6 mice used for the experiments were purchased from the Laboratory Animal Center of Lanzhou University, and the collection of sheep samples were licensed by the slaughterhouse. The in vivo experiments were carried out according to the protocols (2015-03-002) approved by the Institutional Animal Care and Use Committee of Lanzhou University.

## Results

### Efficacy of AA against *E. granulosus* s.s. protoscoleces in vitro

**Figure 1:**
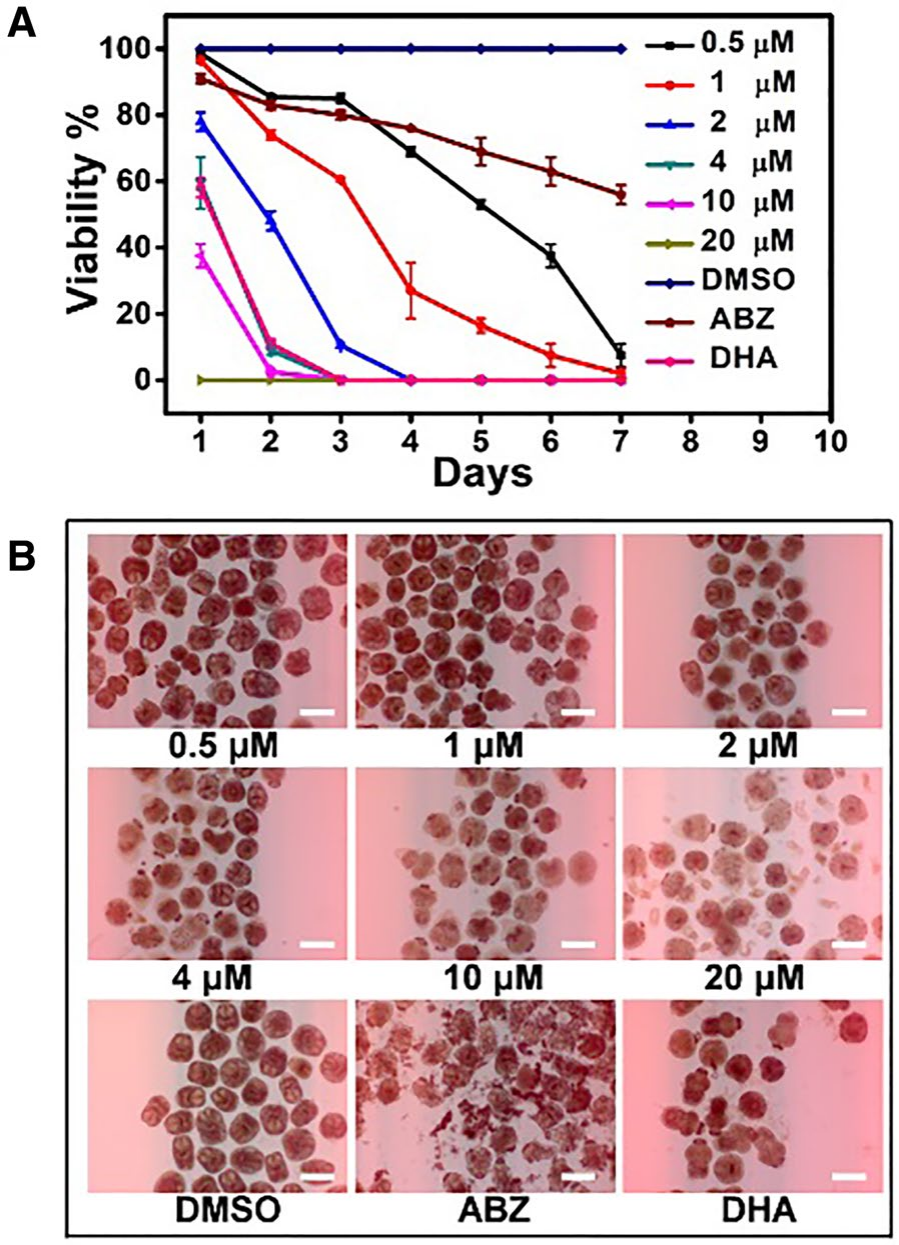
**Efficacy of AA against** ***E. granulosus*** **protoscoleces in vitro. A** Protoscoleces of *E. granulosus* were treated with different concentrations of AA for 7 days, and the viability of the parasites was assessed by trypan blue staining. The equal amount of 40 μM ABZ and 40 μM DHA (positive), as well as 0.1% DMSO (negative) served as controls. **B** The effects of the different drugs on the morphology and structural integrity of protoscoleces were visualized 2 days after treatment without trypan blue staining. The scale bar corresponds to 400 μm.

*Echinococcus granulosus* s.s. protoscoleces were completely exterminated after 24 h of AA (20 μM) treatment and 72 h of DHA (40 μM) treatment. In addition, the mortality of protoscoleces reached 92.5% after 0.5 μM AA treatment on day 7, whereas 56% protoscoleces were still viable on day 7 after treatment with 40 μM of ABZ (Figure 1A). All of these indicated that AA had a higher efficacy than DHA and ABZ on killing *E. granulosus* s.s. protoscoleces. Moreover, the morphological alterations and the mortality rate of protoscoleces on day 2 after treatment with the drugs (before trypan blue staining) were also evident (Figure 1B). The structure of the protoscoleces was markedly altered in the AA-treated group in a dose-dependent manner and a similar result was observed in the trypan blue staining test as well.

### Efficacy of AA against *E. multilocularis* metacestodes in vitro

**Figure 2:**
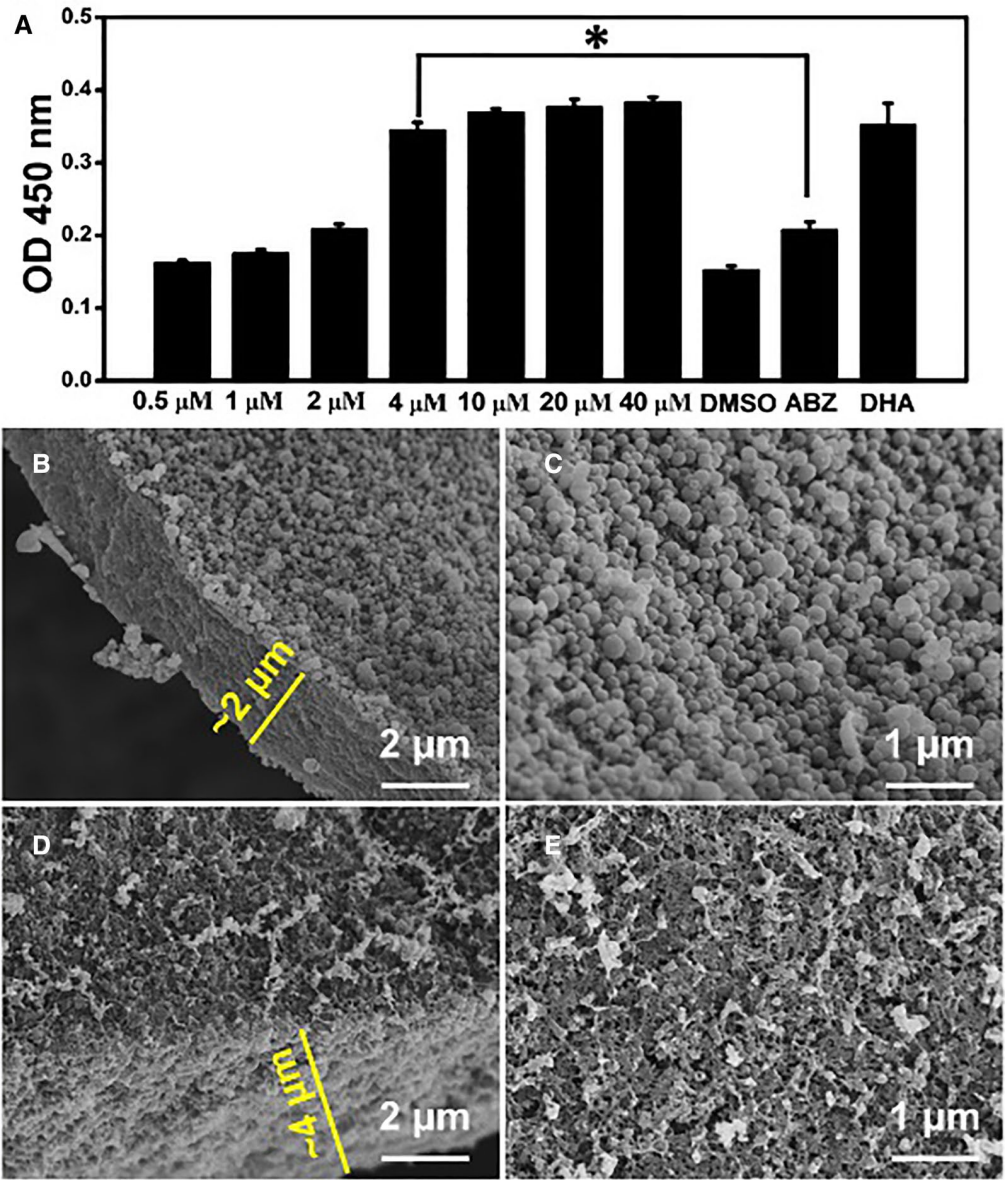
**Efficacy of AA against** ***E. multilocularis*** **metacestodes in vitro. A** *E. multilocularis* metacestodes were treated with different concentrations of AA, 0.1% DMSO, 40 μM ABZ and 40 μM DHA, and alkaline phosphatase activity was detected during the treatments by EmAP assay. *E. multilocularis* metacestodes were exposed to the 0.1% DMSO (**B**, **C**) and 0.5 μM AA (**D**, **E**), and observed by SEM. Asterisks indicate scores obtained using one-way ANOVA in comparison with the ABZ group results.

EmAP activity in the medium supernatant has been used as an indicator assay to demonstrate the loss of viability of drug-treated vesicles for *E. multilocularis* metacestodes [28]. Therefore, we investigated the effects of AA on *E. multilocularis* metacestodes via the EmAP activity assay. EmAP levels were strongly increased as the concentration of AA increased, and reached a plateau at 4 μM on day 7 (Figure 2A). One-way ANOVA analysis indicated that when the concentration of AA was higher than 4 μM, a significant increase in EmAP activity in the AA-treated groups, compared with the ABZ-treated groups, was observed (*p* < 0.05), while no significant differences between the AA- and DHA-treated groups were seen on day 7 (*p* > 0.05). The damage to metacestodes imposed by AA treatment was further demonstrated by SEM, which showed that the AA exhibited a devastating impact on *E. multilocularis* metacestodes, where the major portion of the germinal layer was largely distorted and only tissue residues remained (Figures 2D and E) while the control group of DMSO-treated metacestodes showed no significant damage to the germinal layer (Figures 2B and C). In addition, at the same magnification, the laminated layer in the AA-treated group (Figure 2D) displayed remarkable loose compared with the control group (Figure 2B).

### Efficacy of AA against *Echinococcus* metacestodes in vivo

**Figure 3:**
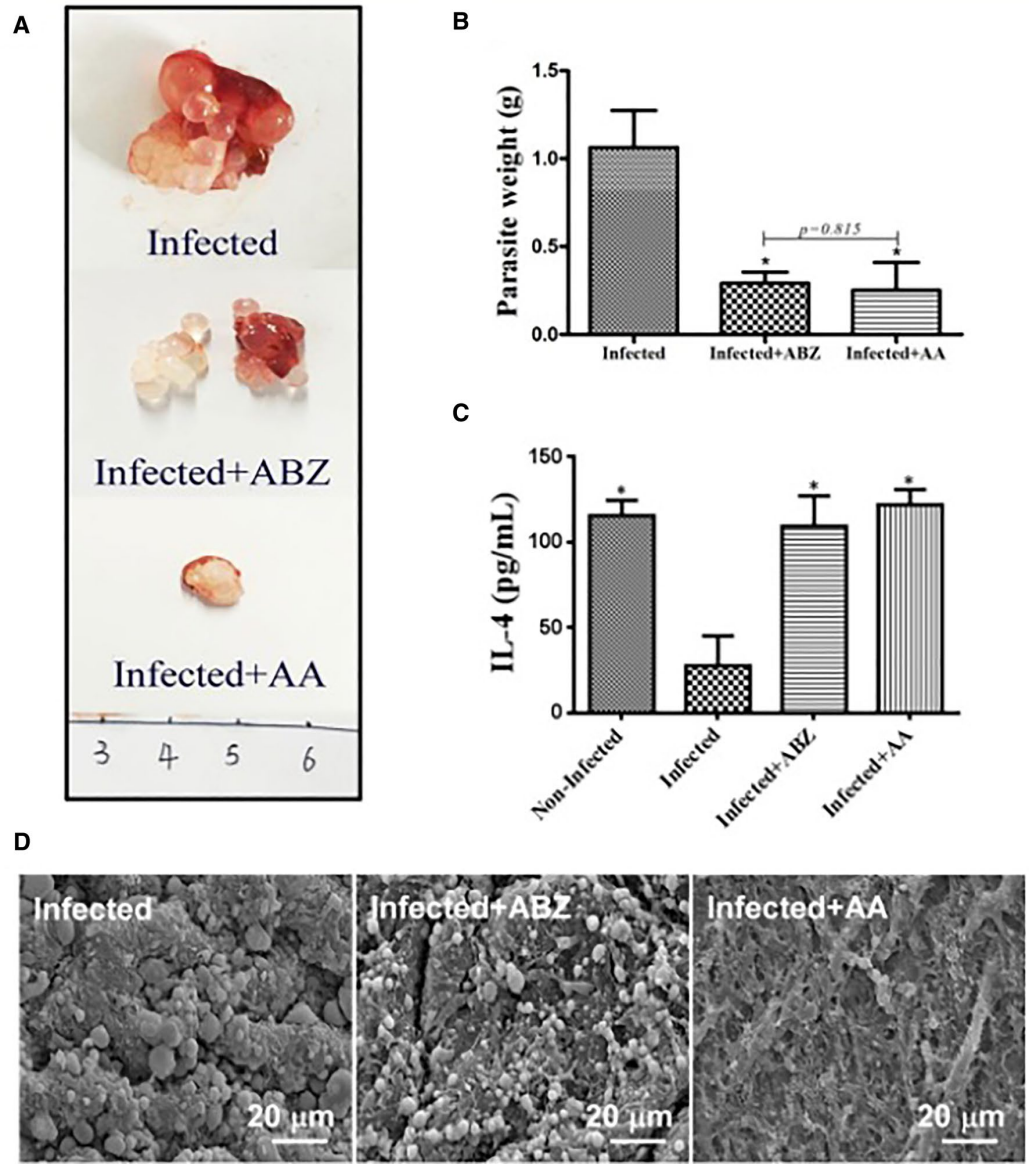
**Efficacy of AA against** ***E. granulosus*** **metacestodes in vivo.** Mice were intraperitoneal injected *E. granulosus* metacestodes for 18 weeks, and then treated with honey/PBS, ABZ or AA orally for 6 weeks. The images (**A**) and weight (**B**) of metacestodes resected from different treatment groups. **C** The level of IL-4 was detected after treatment with ABZ and AA by ELISA. Asterisks indicate scores obtained using one-way ANOVA in relation to the control group. **D** *E. granulosus* metacestodes in different treatment groups were observed by SEM.

**Figure 4:**
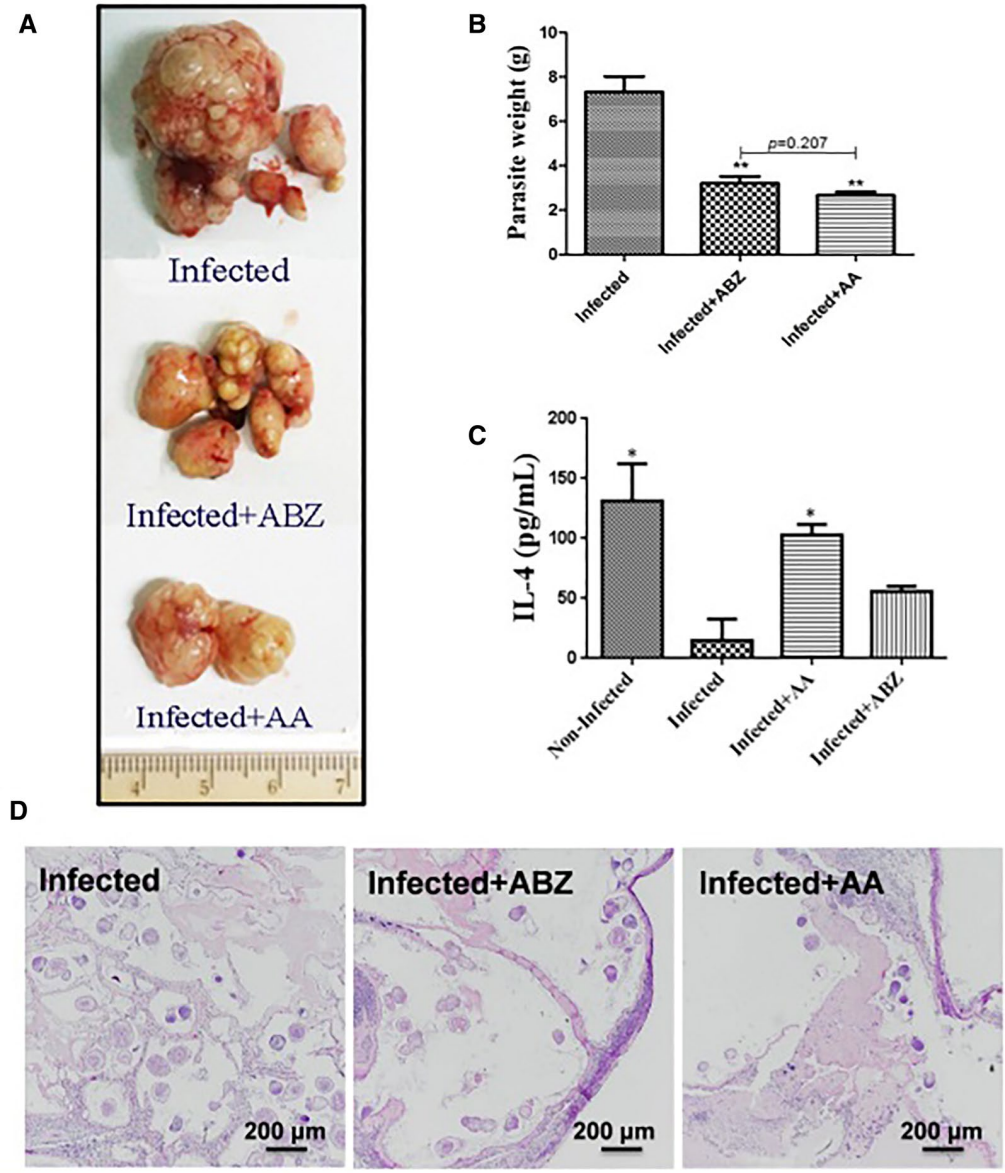
**Efficacy of AA against** ***E. multilocularis*** **metacestodes in vivo.** The images (**A**) and weight (**B**) of metacestodes resected from different treatment groups. **C** The level of IL-4 was detected after treatment with ABZ and AA by ELISA. Asterisks indicate scores obtained using one-way ANOVA in relation to the control group. **D** H&E-staining of *E. multilocularis* metacestodes from different treatment groups.

AA could significantly inhibit *E. granulosus* s.s. metacestodes (Figures 3A and B) and *E. multilocularis* metacestodes (Figures 4A and B) in vivo. We also detected the impact of AA on IL-4 level and found that IL-4 level was significantly increased after AA treatment (Figures 3C and 4C). Typical structures of hydatid cysts are characterized by a clear laminated layer, an intact and densely-packed germinal layer, abundant undifferentiated cells and muscle cells, and the protrusion of the microtriches into the laminated layer. Ultrastructural studies by SEM revealed noticeable changes in the metacestodes from the ABZ or AA-treated *E. granulosus* s.s.—infected mice, including the detachment of the germinal layer from the laminated layer and loss of the standard structure of the germinal layer, especially in the AA- treatment group (Figure 3D). Moreover, H&E staining also indicated that the amount and structure of the protoscoleces were markedly reduced and altered in the ABZ or AA treated *E. multilocularis*-infected mice (Figure 4D). These findings were entirely consistent with the experimental results in vitro presented above that AA has higher anti-*Echinococcus* activities.

### Cytotoxicity of AA on Chang liver cells and HepG2 cells

**Figure 5:**
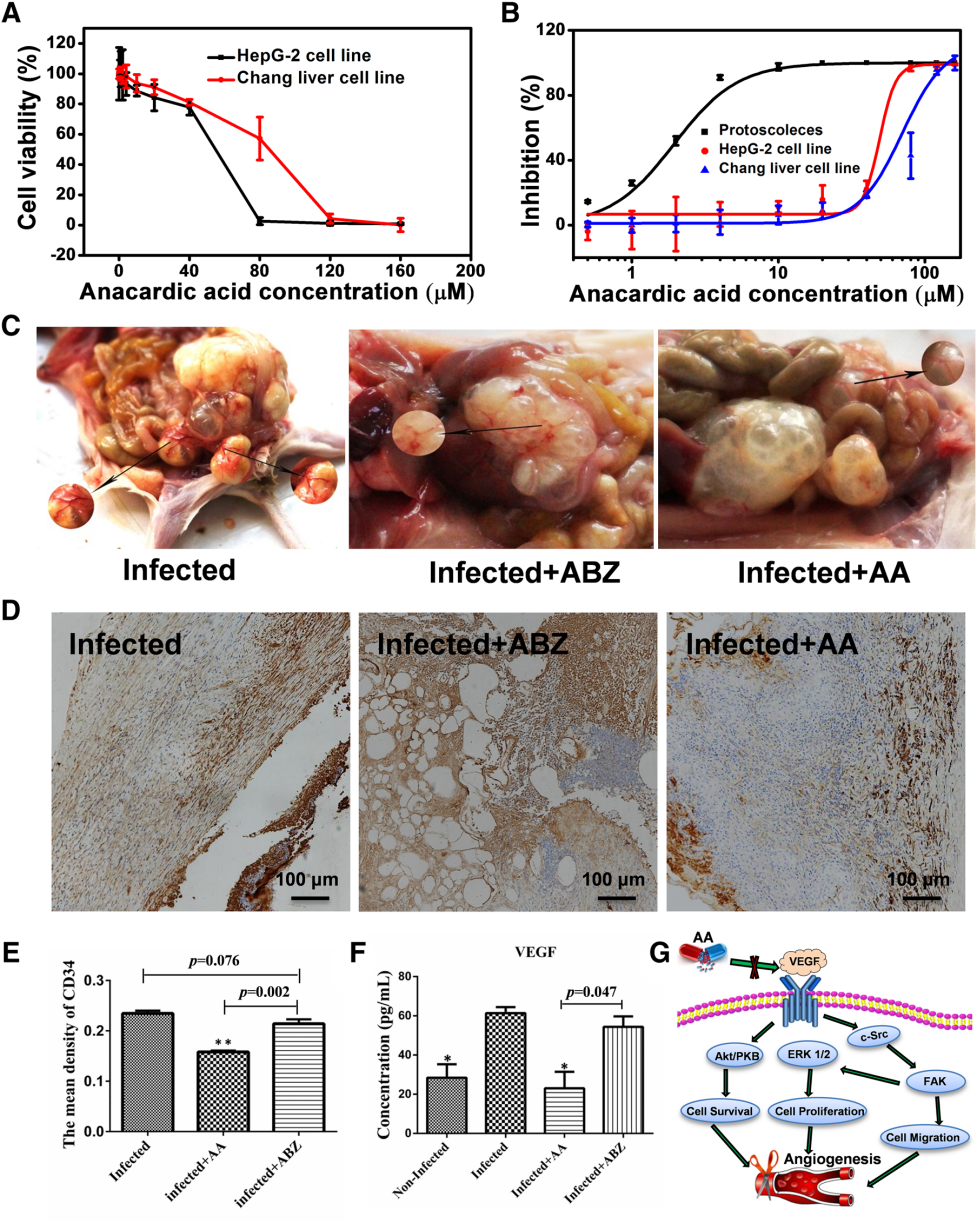
**The cytotoxicity and mechanism of AA against** ***Echinococcus***. **A** Cytotoxicity of AA was measured via Chang liver cell line and HepG2 cell line by MTT assay. **B** The EC_50_ value of AA on protoscolices and the IC_50_ values of AA on HepG2 cells and Chang liver cells were calculated in OriginPro 8. **C** Images of blood vessel of metacestodes in different treatment groups. **D** The sections of the metacestodes from different treatment groups were showed by IHC analysis with anti CD34 antibodies. **E** The mean density of CD34 in different treatment groups were showed, which was calculated by Image-J software. **F** The level of VEGF was measured after treatment with ABZ and AA by ELISA. **G** Schematic representation of the mechanism underlying the AA-mediated inhibition of VEGF-induced angiogenesis.

Secondary effects often limit the clinical application of many drugs. For this reason, the cytotoxicity of AA was assessed using the MTT assay. Our results showed that the effective concentration of AA (20 μM) on metacestodes has no apparent cytotoxicity on the detected cells (Figure 5A). The EC_50_ value of AA for 48 h treatment was 1.9 ± 2 μΜ, and the IC_50_ values of AA on HepG2 cells and Chang liver cells were 49.0 ± 8.0 μM and 70.0 ± 3.0 μM, respectively (Figure 5B). These results suggested that AA was a promising candidate drug against echinococcosis with high activity and without apparent cytotoxicity.

### AA against *E. multilocularis* metacestodes through inhibiting angiogenesis

To understand the mechanism of how AA suppresses metacestodes, we investigated whether AA could affect angiogenesis in vivo. In the control group, the viable metacestodes were surrounded by more blood vessels compared with the ABZ- and AA-treated groups (Figure 5C). To further confirm whether AA could affect angiogenesis in vivo, the expression of CD34 and the level of VEGF were detected. We stained the metacestodes sections with specific anti-CD34 antibodies. As shown in Figures 5D and E, the blood vessel area as indicated by CD34 immunohistochemical staining in AA-treated group reduced significantly when compared with the control group. Furthermore, we found that the level of VEGF was obviously suppressed in the AA-treated group compared with the infected control group.

## Discussion

Anti-hydatid drugs such as benzimidazole derivatives are not adequate at present owing to their limited effectiveness in killing parasites stem cells [28], poor intestinal absorption, severe adverse reactions, and drug resistance [32]. For this reason, there is an urgent need to identify novel, more effective drugs. In this study, the in vivo and in vitro efficacy of AA against both CE and AE, the two most popular echinococcosis in human, were evaluated. AA effectively inhibited the growth of *Echinococcus* in vitro and in vivo and even more efficacious than ABZ and DHA in vitro. It has been reported that DHA has a strong activity against *E. granulosus* protoscoleces and *E. multilocularis* metacestodes. However, this drug only decreased the wet weight of metacestodes slightly in an in vivo mouse model and thus implied that DHA could be inefficacious or less efficacious in vivo [33]. Therefore, in this research, DHA was only used as a positive control for in vitro experiment and the in vivo experiment of AA was conducted with ABZ as the positive control against both *E. granulosus* s.s. protoscoleces and *E. multilocularis* metacestodes. Besides, the mebendazole is active against the protoscoleces and germinal cells, while the reduction of the metacestodes in vivo is not significant [34]. Fortunately, AA exhibited a high anti-hydatid activity not only in vitro, which had been described above, but also in vivo, where it inhibited the growth of *Echinococcus* metacestode as efficiently as ABZ. An interesting finding from our experiment was the remarkable loosened and thickened laminated layer resulted from AA, which was not only the result of AA on the cysts or vesicles, but also a promoter that would further facilitate AA (or other anti-hydatid drugs) passing into the cysts or vesicles and further strengthen the treatment effect. To the best of our knowledge, this is first time to find and describe the impact on the laminated layer of hydatid cyst from a drug and the effect of AA on the laminated layer of hydatid cyst. All the findings strongly suggest that AA is a promising candidate drug for the effective treatment of echinococcosis.

It is well known that cytokines play an important role in the host immune response to parasite infection, and correspondingly the therapeutic effects of anti-parasite agents can be evaluated by monitoring the change of the serum cytokines level, e.g. the level of IL-4 in echinococcosis [8]. In this study, the results displayed that similarly in both the CE and AE infected mice, the level of IL-4 of the ABZ- and AA-treated groups increased significantly compared with the infected control group, which is in agreement with our previous report [8]. It proved that the increase of IL-4 is an indicator in favor of the host at the late stage of infection.

The anti-tumoral activity of AA has been elucidated through inhibiting histone acetyltransferases [23], nuclear factor-kappa B [35], and tumor angiogenesis by targeting Src/FAK/Rho GTPases Signaling Pathway [36]. However, how AA acts against echinococcosis still remains unknown. Angiogenesis and the production of angiogenic factors are fundamental for tumor progression in the form of growth, invasion, and metastasis [37]. CD34 is well known as an endothelial marker, and it presents positive staining in physiologic and pathologic vessels, and is also treated as an ideal marker for microvascular density studies because of its good immunoreactivity [38]. Our study indicated that the density of CD34 in the AA-treated group presented a significant reduction compared with the control group, indicating that AA significantly inhibited angiogenesis in metacestodes. The VEGF is widely expressed in many cancers and is a critical component of tumor angiogenesis [39]. VEGF signaling regulating angiogenesis is mainly mediated by activation of its downstream signaling targets, including Src/FAK pathway [40] and Akt/PKB pathway [41]. In addition, it has been observed that Src kinase group plays a major role in VEGF-mediated angiogenesis by AA to cancer cells [36]. At here, our study indicated that the level of VEGF in AA-treated group significantly reduced compared with the control group, and therefore the possible mechanism of AA inhibiting angiogenesis in *Echinococcus* metacestode was mainly through the inhibition of VEGF-induced Src/FAK and Akt/PKB expression (Figure 5G).

The in vivo toxicity of AA has been evaluated and discovered no obvious adverse effects in BALB/c mice with a dosage no more than 300 mg/kg [42]. The result is consistent with that in vitro using the MTT assay in our research. In addition, following 48 h of treatment with AA, the EC_50_ value of AA on protoscolices was 1.9 ± 2 μM, while the IC_50_ values on HepG2 cells and Chang liver cells were 49.0 ± 8.0 μM and 70.0 ± 3.0 μM, respectively. These results demonstrated that effective treatment concentrations of AA on *Echinococcus* did not significantly affect human cells.

Taken together, our results show that in vitro and in vivo, AA exhibits profound activities against *Echinococcus* with no obvious toxicity. It is worth mentioning that inhibiting VEGF-induced angiogenesis is might be a new drug target and a possible mechanism of AA against *Echinococcus*. Therefore, the great potential exhibited by AA could make it an ideal candidate for the prevention and treatment of echinococcosis.
